# Supplementary figures and images for: Dopaminergic drug treatment remediates exaggerated cingulate prediction error responses in obsessive-compulsive disorder
Source: Psychopharmacology (Berl). 2019 Jun 14;236(8):2325–36. doi: 10.1007/s00213-019-05292-2 (PMC6695357; doi:10.1007/s00213-019-05292-2)

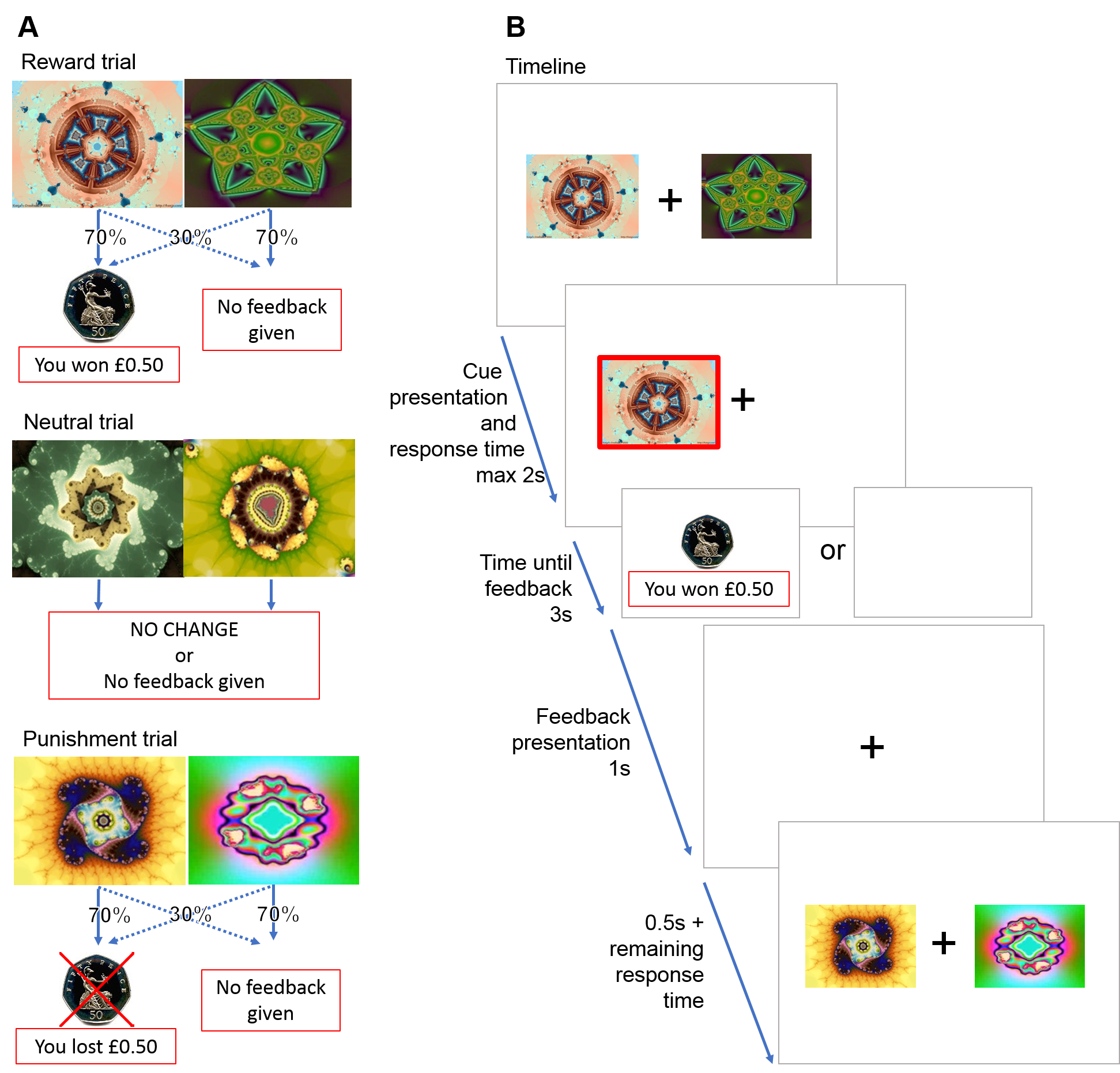

Supplement: Supplementary file 1 — (PNG 1962 kb) [file 213_2019_5292_MOESM1_ESM.png]

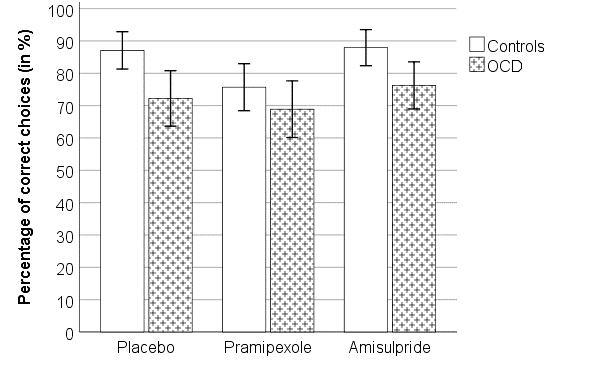

Supplement: Supplementary file 2 — (PNG 14 kb) [file 213_2019_5292_MOESM2_ESM.png]

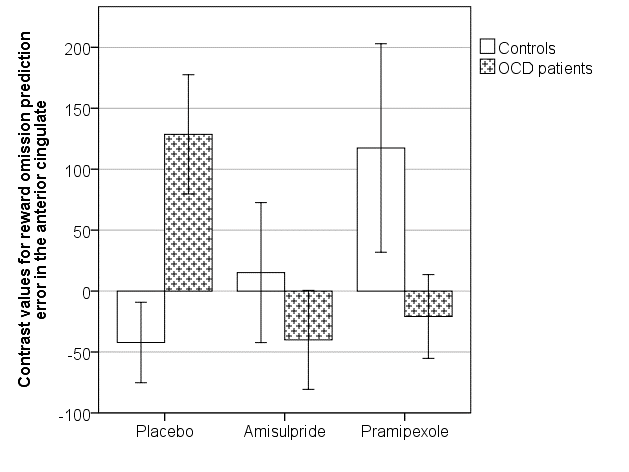

Supplement: Supplementary file 3 — (PNG 14 kb) [file 213_2019_5292_MOESM3_ESM.png]

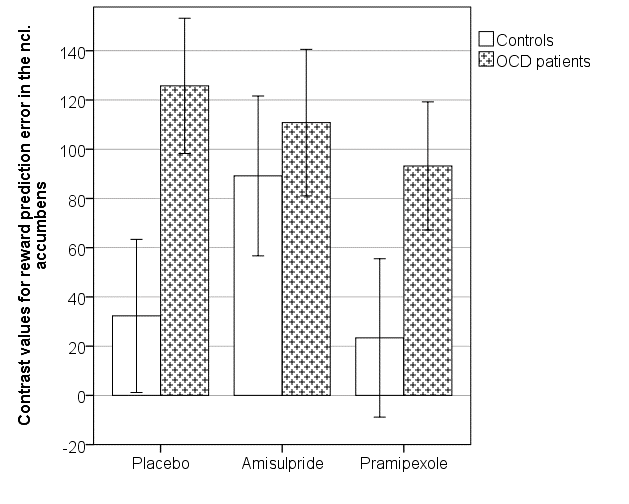

Supplement: Supplementary file 4 — (PNG 20 kb) [file 213_2019_5292_MOESM4_ESM.png]

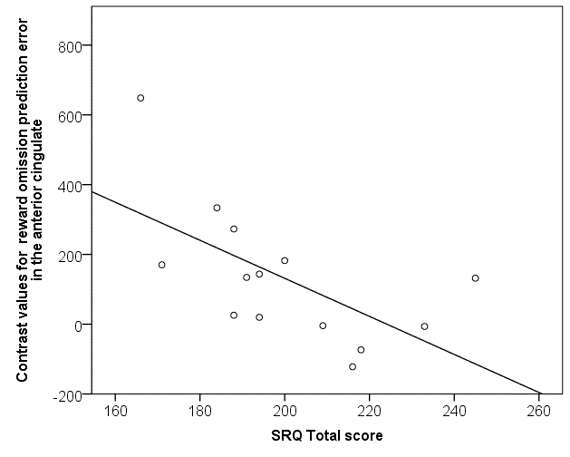

Supplement: Supplementary file 5 — (PNG 14 kb) [file 213_2019_5292_MOESM5_ESM.png]
